# Trends in Timing and Provision of Pediatric Cochlear Implant Care During COVID‐19

**DOI:** 10.1002/oto2.37

**Published:** 2023-02-22

**Authors:** Kimberley S. Noij, Emily Y. Huang, Jonathan Walsh, Francis X. Creighton, Deepa Galaiya, Stephen P. Bowditch, C. Matthew Stewart, Carolyn M. Jenks

**Affiliations:** ^1^ Department of Otolaryngology–Head and Neck Surgery Johns Hopkins University School of Medicine Baltimore Maryland USA

**Keywords:** COVID‐19, pediatric cochlear implantation, rehabilitation

## Abstract

**Objectives:**

To identify trends in timing of pediatric cochlear implant (CI) care during COVID‐19.

**Study Design:**

Retrospective cohort.

**Setting:**

Tertiary care center.

**Methods:**

Patients under 18 years of age who underwent CI between 1/1/2016 and 2/29/2020 were included in the pre‐COVID‐19 group, and patients implanted between 3/1/2020 and 12/31/2021 comprised the COVID‐19 group. Revision and sequential surgeries were excluded. Time intervals between care milestones including severe‐to‐profound hearing loss diagnosis, initial CI candidacy evaluation, and surgery were compared among groups, as were the number and type of postoperative visits.

**Results:**

A total of 98 patients met criteria; 70 were implanted pre‐COVID‐19 and 28 during COVID‐19. A significant increase in the interval between CI candidacy evaluation and surgery was seen among patients with prelingual deafness during COVID‐19 compared with pre‐COVID‐19 (*µ* = 47.3 weeks, 95% confidence interval [CI]: 34.8‐59.9 vs *µ* = 20.5 weeks, 95% CI: 13.1‐27.9; *p* < .001). Patients in the COVID‐19 group attended fewer in‐person rehabilitation visits in the 12 months after surgery (*µ* = 14.9 visits, 95% CI: 9.7‐20.1 vs *µ* = 20.9, 95% CI: 18.1‐23.7; *p* = .04). Average age at implantation in the COVID‐19 group was 5.7 years (95% CI: 4.0‐7.5) versus 3.7 years in the pre‐COVID‐19 group (95% CI: 2.9‐4.6; *p* = .05). The time interval between hearing loss confirmation and CI surgery was on average 99.7 weeks for patients implanted during COVID‐19 (95% CI: 48.8‐150) versus 54.2 weeks for patients implanted pre‐COVID (95% CI: 39.6‐68.8), which was not a statistically significant difference (*p* = .1).

**Conclusion:**

During the COVID‐19 pandemic patients with prelingual deafness experienced delays in care relative to patients implanted before the pandemic.

Hearing loss is highly prevalent in children, affecting nearly 1 in 5 children by age 18.^1^ Cochlear implantation (CI) is recommended for children with severe‐to‐profound sensorineural hearing loss (SNHL). Early implantation is indicated, as the first 2 years of life are critical for speech and language development.^2^ Studies have found that early CI in prelingual patients has significant effects on speech, language, functional and social outcomes.^3^, ^4^, ^5^, ^6^

The Joint Committee on Infant Hearing recommends that congenital hearing loss is diagnosed by 3 months of age, and a hearing aid trial is started within 1 month of confirmation.^7^ If no improvement is evident with hearing aids and SNHL is profound with a pure tone average of 90 dB HL or greater in the better hearing ear, implantation should be performed ideally by 12 months of age.^2^

CI candidacy evaluation includes the following components: (1) medical evaluation, (2) audiologic assessment, (3) speech and language assessment, (4) patient and family counseling and (5) imaging.^8^ This process requires multiple appointments that are completed over a variable amount of time based on patient factors, family availability, and hospital resources.^9^ The COVID‐19 pandemic has delayed medical and surgical care in children due to safety precautions, workforce shortages, and resource limitations.^10^, ^11^ Hearing screening programs, outpatient appointments, and imaging needed for workup of CI candidacy, surgeries, and rehabilitation appointments have been limited during the pandemic.^12^, ^13^ Parents of CI recipients reported delays in hearing health services due to inability to follow up with their centers during the pandemic.^14^

Previous work assessing delays in surgical care during COVID‐19 showed that otolaryngology was among the most affected surgical specialties with a decrease of 30‐40% in monthly case volumes, and that within otolaryngology, otology and general/pediatric otolaryngology were most affected.^15^, ^16^ At our institution, a tiered system was created to determine surgical priority. Pediatric CI surgery was considered tier 1 (highest priority) based on the limited window for speech acquisition and need for early intervention.^2^ Despite this and other attempts to avoid delays in critical surgical care, we hypothesize that pediatric CI candidates experienced delays in care during COVID‐19.

This study aims to identify trends and possible delays in the timing of pediatric CI pre‐ and postoperative care among patients who underwent CI during the COVID‐19 pandemic compared with a similar cohort of patients who underwent CI in the years pre‐COVID‐19 at a single tertiary care academic medical center. Findings could help identify gaps in pediatric CI care and areas for improvement.

## Methods

Approval for this study was obtained from the Institutional Review Board at Johns Hopkins University. Pediatric patients (age <18 years) who underwent CI at a single tertiary care center between January 1, 2016 and December 31, 2021 were retrospectively reviewed. Patients were divided into 2 groups based on the date of CI surgery. Patients implanted between January 1, 2016 and February 29, 2020 were included in the pre‐COVID‐19 group, while patients implanted between March 1, 2020 and December 31, 2021 were included in the COVID‐19 group. Revision and second‐side CI surgeries were excluded.

The following data were retrospectively reviewed: age at implantation, sex, parent‐ reported race, ethnicity, whether an interpreter was present during visits, if patients passed newborn hearing screen, prelingual deafness, and laterality of CI. Dates of severe‐to‐profound hearing loss confirmation, initial CI candidacy evaluation, hearing aid trial, imaging, and CI surgery were collected. Patients whose hearing aid use predated confirmation of severe‐to‐profound hearing loss were excluded from analysis of intervals involving hearing aid trial date. Postoperative dates were collected for CI activation and initial rehabilitation visit. Time intervals between care milestones were compared for the pre‐COVID‐19 and COVID‐19 groups. The number and type (virtual versus in person) of postoperative rehabilitation visits in the first 6 and 12 months after surgery were compared between groups.

Statistical analysis was performed using SPSS (version 25.0). Demographic variables for the pre‐COVID‐19 and COVID‐19 group were compared using an independent samples *t*‐test for age and *χ* ^2^ tests for all others. Analyses of variance (ANOVA) tests were used to determine the effect of pre‐COVID‐19 versus COVID‐19 groups on these variables. The following additional factors were included in the ANOVA: race, ethnicity, need for an interpreter, prelingual deafness and uni‐ versus bilateral simultaneous implantation. Factors without a significant effect were removed from the models for subsequent analyses. *p* Values of <.05 were considered statistically significant. For significant interactions, post‐hoc pairwise comparisons were performed using a Bonferroni adjustment for multiple comparisons.

## Results

### Patient Characteristics

After excluding second‐side sequential and revision surgery cases, a total of 98 primary pediatric CI surgeries were performed between January 1, 2016 and December 31, 2021. There were 68 unilateral and 30 bilateral simultaneous implantations. Of these, 70 surgeries were performed pre‐COVID‐19 and 28 during COVID‐19. Characteristics of patients who underwent their first CI surgery before March 1, 2021 (pre‐COVID‐19 group) and after (COVID‐19 group) are presented in Table 1. No significant differences were found between the pre‐COVID‐19 and COVID‐19 groups for sex, race, ethnicity, need for an interpreter, passing of newborn hearing screen, prelingual deafness or laterality in CI. Patients in the COVID‐19 group had an average age of 5.7 years at the time of their first CI surgery (95% confidence interval [CI]: 4.0‐7.5) compared with 3.7 years in the pre‐COVID‐19 group (95% CI: 2.9‐4.6). This difference was not statistically significant (*p* = .05).

**Table 1:** Demographics of patients in the pre‐COVID‐19 and COVID‐19 groups

	Pre‐COVID‐19 (n = 70)	COVID‐19 (n = 28)	*p* Value
Sex, n (%)			.1
Female	35 (50.0)	19 (67.9)	
Male	35 (50.0)	9 (32.1)	
Race, n (%)			
White	26 (37.1)	16 (57.1)	.07
Black or African American	7 (10.0)	5 (17.9)	.4
Native Hawaiian or Pacific Islander	1 (1.4)	0 (0)	.5
Asian	10 (14.3)	2 (7.0)	.3
American Indian or Alaskan Native	0 (0)	1 (3.6)	.1
More than one race	1 (1.4)	1 (3.6)	.5
Ethnicity, n (%)			.9
Not Hispanic or Latino	57 (81.4)	23 (82.1)	
Hispanic or Latino	13 (18.6)	5 (17.9)	
Interpreter present, n (%)	23 (31.4)	5 (17.6)	.2
Passed newborn hearing screen, n (%)	41 (58.6)	15 (53.6)	.9
Prelingual deafness, n (%)	54 (77.1)	19 (67.8)	.3
Implanted side, n (%)			
Left	20 (28.6)	12 (42.9)	.2
Right	26 (37.1)	10 (35.7)	.9
Simultaneous bilateral	24 (34.3)	6 (21.4)	.2

*Note*: *χ* ^2^ tests were used to evaluate for demographic differences between the pre‐COVID and COVID groups.

### Preoperative Care Milestones

**Figure 1:**
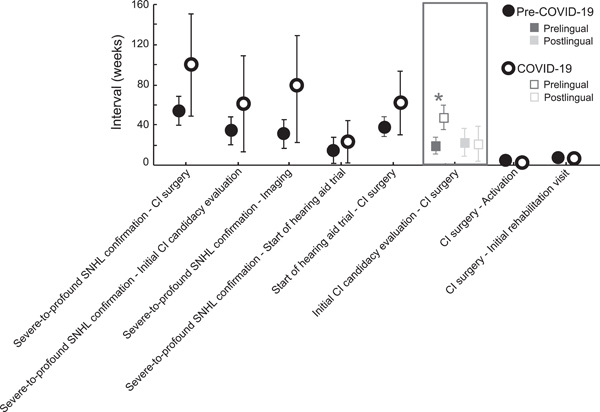
Means and 95% confidence intervals are displayed. Prelingual versus postlingual deafness were analyzed and displayed separately (boxed) due to a significant interaction. The asterisk indicates a statistically significant difference.

Time intervals between pre‐ and postoperative care milestones are depicted in Figure 1. The interval in weeks between confirmation of severe‐to‐profound SNHL and first CI candidacy evaluation was not statistically significantly different in the COVID‐19 group (*µ* = 61.1 weeks, 95% CI: 13.3‐108.9) compared with the pre‐COVID‐19 group (*µ* = 34.2 weeks, 95% CI: 20.3‐48.1; *p* = .2). Patients with postlingual deafness had a longer interval between severe‐to‐profound SNHL confirmation and first CI candidacy evaluation regardless of pre‐ or COVID‐19 timing (prelingual deafness *µ* = 33.2 weeks, 95% CI: 12.8‐53.6 vs postlingual deafness *µ* = 95.6 weeks, 95% CI: 59.9‐131.4; *p* = .002). Interval from confirmation of severe‐to‐profound SNHL to first CI candidacy evaluation was not significantly different among race (*p* = .7), ethnicity (*p* = .8), need for an interpreter (*p* = .5) or uni‐ versus bilateral simultaneous implantation (*p* = .6) groups.

The time from severe‐to‐profound SNHL confirmation to imaging was also longer for patients with postlingual deafness compared with patients with prelingual deafness regardless of COVID‐19 group (prelingual deafness *µ* = 38.1 weeks, 95% CI: 15.9‐60.3 vs postlingual deafness *µ* = 104.3 weeks, 95% CI: 66.5‐142.2; *p* = .002). This interval was not significantly different among COVID‐19 (*p* = .1) race (*p* = .2), ethnicity (*p* = .6), need for an interpreter (*p* = .4) or uni‐ versus bilateral simultaneous implantation (*p* = .9) groups.

There was no significant difference in time interval between severe‐to‐profound SNHL confirmation and start of hearing aid trial (pre‐COVID‐19 *µ* = 14.9 weeks, 95% CI: 1.9‐27.9 vs COVID‐19 *µ* = 23.3 weeks, 95% CI: 2.2‐44.4; *p* = .3), with no significant effect of race (*p* = .9), ethnicity (*p* = .8), need for an interpreter (*p* = .2), prelingual deafness (*p* = .2) or uni‐ versus bilateral simultaneous implantation (*p* = .9). Similarly, there was no significant difference in time interval between hearing aid trial and surgery (pre‐COVID‐19 *µ* = 38.5 weeks, 95% CI: 28.1‐48.8 vs COVID‐19 *µ* = 61.2 weeks, 95% CI: 28.5‐93.9; *p* = .2). Time interval between hearing aid trial and surgery was not significantly different among race (*p* = .7), ethnicity (*p* = .5), need for an interpreter (*p* = .9), prelingual deafness (*p* = .7) and uni‐ versus bilateral simultaneous implantation (*p* = .06) groups.

Regarding the interval between initial CI candidacy evaluation to CI surgery, there was a significant interaction between COVID‐19 groups (pre‐COVID‐19 vs COVID‐19) and prelingual deafness (prelingual vs postlingual deafness; *p* = .04). This interval was significantly prolonged for prelingually deaf patients in the COVID‐19 group (COVID‐19 *µ* = 47.3 weeks, 95% CI: 34.8‐59.9 vs pre‐COVID‐19 *µ* = 20.5 weeks, 95% CI: 13.1‐27.9; *p* < .001), while there was no significant difference in this interval for postlingually deaf patients (pre‐COVID‐19 *µ* = 22.9 weeks, 95% CI: 8.9‐37.1 vs COVID‐19 *µ* = 21.2 weeks, 95% CI: 2.9‐39.5; *p* = .9). In addition, prelingually deaf patients in the COVID‐19 group had a significantly longer interval between initial CI candidacy evaluation to surgery compared with patients with postlingual deafness (*p* = .02). This interval was not significantly different among race (*p* = .3), ethnicity (*p* = .4), need for an interpreter (*p* = .7) or uni‐ versus bilateral simultaneous implantation (*p* = .08) groups.

The cumulative increase in time between preoperative care milestones resulted in an average overall interval between severe‐to‐profound hearing loss confirmation and CI surgery of 99.7 weeks for the COVID‐19 group (95% CI: 48.8‐150) compared with 54.2 weeks in the pre‐COVID‐19 group (95% CI: 39.6‐68.8). This difference did not reach statistical significance (*p* = .1, Figure 1). This interval was not significantly different among race (*p* = .6), ethnicity (*p* = .5), need for an interpreter (*p* = .5), prelingual deafness (*p* = .2) or uni‐ vs bilateral simultaneous implantation (*p* = .3) groups.

### Postoperative Care Milestones

Means and 95% CIs of the postoperative time intervals are displayed in Figure 1. All patients underwent activation within 7 weeks after implantation, although the time between surgery and activation was significantly shorter for the COVID‐19 group (*µ* = 3.9 weeks, 95% CI: 3.6‐4.2) compared with the pre‐COVID‐19 group (*µ* = 4.4 weeks, 95% CI: 4.3‐4.5; *p* = .001). Patients requiring an interpreter had a longer interval between surgery and activation (need for interpreter *µ* = 4.4 weeks, 95% CI: 4.2‐4.7 vs no need for interpreter *µ* = 4.1 weeks, 95% CI: 3.9‐4.2; *p* = .02). This interval was not significantly different among race (*p* = .6), ethnicity (*p* = .5), prelingual deafness (*p* = .4) and uni‐ versus bilateral simultaneous implantation (*p* = .6) groups.

In the pre‐COVID‐19 group, 15 patients (21.4%) did not attend rehabilitation visits at Johns Hopkins (10 received rehabilitation elsewhere and 5 were unknown). In the COVID‐19 group, 5 patients (17.8%) did not attend rehabilitation visits at Johns Hopkins (2 received rehabilitation elsewhere and 3 were unknown). These patients were excluded from the analysis of timing and frequency of rehabilitation visits. There was no significant difference in time interval between surgery and initial rehabilitation visit between the pre‐COVID‐19 (*µ* = 8.7 weeks, 95% CI: 7.9‐9.5) and COVID‐19 groups (*µ* = 7.5 weeks, 95% CI: 6.8‐8.2; *p* = .1). This interval was not significantly different among race (*p* = .9), ethnicity (*p* = .9), prelingual deafness (*p* = .9), need for an interpreter (*p* = .9) and uni‐ versus bilateral simultaneous implantation (*p* = .7) groups.

Patients in the pre‐COVID‐19 group attended more in person rehabilitation visits in the first 6 months (*µ* = 11.0 visits, 95% CI: 9.6‐12.5 vs *µ* = 8.3 visits, 95% CI: 6.2‐10.4; *p* = .04) and 12 months after surgery (*µ* = 20.9 visits, 95% CI: 18.1‐23.7 vs *µ* = 14.9 visits, 95% CI: 9.7‐20.1; *p* = .04) compared with the COVID‐19 group. There were no significant differences in total postoperative rehabilitation visits (including virtual visits) in the first 6 months (pre‐COVID‐19 *µ* = 11.7 visits, 95% CI: 10.3‐13.0 vs COVID‐19 *µ* = 9.6 visits, 95% CI: 7.4‐11.7; *p* = .09) and 12 months after surgery (pre‐COVID‐19 *µ* = 22.7 visits, 95% CI: 19.6‐25.7 vs COVID‐19 *µ* = 18.8 visits, 95% CI: 13.9‐23.8; *p* = .2). Number of postoperative rehabilitation visits was not significantly different among race, ethnicity, prelingual deafness and uni‐ versus bilateral implantation groups.

Virtual rehabilitation visits only occurred after COVID‐19 started. In the first 6 months after surgery, 8 patients attended at least one virtual rehabilitation appointment (*µ* = 7.5 visits, 95% CI: 4.3‐10.8). In the first 12 months after surgery, 11 patients attended at least one virtual rehabilitation appointment (*µ* = 13.9, 95% CI: 7.7‐20.1). Utilization of virtual rehabilitation was not significantly different among race, ethnicity, need for interpreter, prelingual deafness or uni‐ versus bilateral implantation groups.

## Discussion

The COVID‐19 pandemic disrupted the provision of medical care in far‐reaching ways. Delays in essential and elective surgical care were realized as medical resources were allocated towards caring for patients with COVID‐19.^17^, ^19^ To our knowledge, this is the first study to examine the impact of the COVID‐19 pandemic on pediatric CI care. Overall, there was a trend toward a longer time interval between SNHL confirmation to CI surgery during COVID‐19, however this effect was not statistically significant and could not be attributed to one specific preoperative milestone such as time to imaging, first hearing aid trial or first CI candidacy evaluation. When the COVID‐19 pandemic began, our institution was quick to recognize the importance of timely CI in pediatric patients as demonstrated by its “tier 1” status in a system that was created in an attempt to avoid delays in essential care. Multi‐disciplinary CI team meetings continued, virtually, throughout the pandemic, which helped to drive the preoperative process along for pediatric patients who continued to receive proactive follow up from a multidisciplinary team. It seems that these anticipatory measures were largely successful in limiting delays in pediatric CI care. Despite these efforts, however, patients with prelingual deafness experienced a significant prolongation in interval between initial CI candidacy evaluation to CI surgery during COVID‐19, while there was no significant difference in this interval for patients with postlingual deafness.

Patients with prelingual deafness are particularly vulnerable to delays in CI because of a critical window for speech and language development.^20^, ^21^, ^22^, ^23^ On average, the interval between CI candidacy evaluation to CI surgery was 6 months longer for patients with prelingual deafness that were implanted during COVID‐19 compared with those implanted pre‐COVID. Numerous studies have demonstrated that timing of CI impacts speech, language, functional and social outcomes in children.^2^, ^24^, ^25^, ^26^, ^27^, ^28^, ^29^ Direct comparisons of children implanted earlier versus later consistently demonstrate superior outcomes among children implanted at an early age.^24^, ^25^, ^26^, ^27^, ^28^, ^30^, ^31^ Implantation before 12 months of age results in language reception that is on par with normal‐hearing ears and is improved compared with children implanted between 12 and 24 months.^30^, ^31^, ^32^ All together, these data show that a delay on the order of months can have a significant impact on a child's outcomes and highlight the clinical significance of delays in care observed during the COVID‐19 pandemic.

Although patients with preoperative language had a longer interval between SNHL confirmation and first CI candidacy evaluation regardless of COVID‐19 era, this group was less affected by delays during COVID‐19. It is possible that the patients with postlingual deafness had improved access to care during COVID‐19 because of pre‐existing relationships with audiologists and surgeons on the CI team. In addition, some may have completed parts of the required presurgical work‐up prior to COVID‐19.

The range of intervals between SNHL confirmation and CI surgery was wide in both the pre‐COVID‐19 and COVID‐19 groups, with 95% CI of 39.6 to 68.8 weeks and 48.8 to 150 weeks, respectively. DeVries et al^33^ reported a similarly wide range of intervals between SNHL confirmation and CI evaluation (mean 54.9 weeks, standard error of the mean 11.2 weeks, calculated 95% CI: 32.9‐76.8 weeks). Patients may have multiple reasons why they would delay CI workup, regardless of COVID‐19, such as hesitancy toward CI, delayed acceptance of hearing loss, financial restraints, and problems with referrals to another professional after initial consultation.^34^ Variability in these factors likely contributes to the wide range of intervals between care milestones seen among CI patients. Variability in families' safety concerns and willingness to engage in care during the COVID‐19 pandemic may have contributed to the even wider range of intervals observed in this study. Compared with a prior study by Devries et al,^33^ who reported an average time between candidacy identification and implantation of about 1 year, the pre‐COVID‐19 group had a comparable interval and the COVID‐19 group had a longer interval between severe to profound SNHL confirmation and CI candidacy confirmation.

Although no significant effects of race or need for an interpreter were found on time intervals between preoperative care milestones, the percentage of white patients undergoing CI increased from 37.1 in the pre‐COVID‐19 group to 57.1% in the COVID‐19 group, and need for an interpreter decreased from 31.4 to 17.6%, suggesting possible care disparities for non‐white and non‐native English speakers. Race, socioeconomic status, insurance type and language have previously been identified as barriers to CI.^2^, ^35^, ^36^, ^37^ The percentage of non‐white pediatric CI patient across the United States has increased between 1997 (26.7%) to 2012 (41.1%). Historically, white and Asian/Pacific Islander patients have had higher implantation rates compared with Hispanic and black patients, highlighting general racial discrepancy with CI.^36^ This trend was mirrored in Maryland with data obtained from the 2015 Census Bureau.^38^ During COVID‐19, the decrease in percentage of non‐white and non‐native English speakers may reflect worsening disparities.

Contrary to the finding of increased time between preoperative care milestones, the time between surgery and activation was shorter in the COVID‐19 group compared with the pre‐COVID‐19 group, on average by 0.5 week. This reflects a shift toward earlier activation at our institution and is unlikely to be a clinically significant difference. All patients underwent activation within 7 weeks after implantation, and no major delays were observed. At our institution, surgery and activation are scheduled concurrently, following a standardized timeline that likely helped mitigate systemic delays in care during the COVID‐19 era.

The initial postoperative rehabilitation visit was not significantly delayed in the COVID‐19 group, and there were no significant differences in the total number of visits between pre‐COVID‐19 and COVID‐19 groups at 6 and 12 months after surgery. Patients in the COVID‐19 group attended fewer in‐person rehabilitation visits in the first 6 and 12 months after surgery compared with the pre‐COVID‐19 group, however. Recent studies reported that satisfaction and hearing outcomes for virtual rehabilitation and CI mapping were comparable to those expected following in‐person visits, though data regarding the effectiveness of virtual visits remains limited.^39^, ^40^

We acknowledge several limitations to our study. It is possible that the size of the study group was too small to detect statistically significant differences in care milestones. Furthermore, subgroups of interest in this study (race, ethnicity, need for an interpreter) were small, and it is possible that significant effects of these variables were not evident due to sample size. Speech and language outcomes were not examined due to short follow‐up times as of the date of writing, limitations related to patient age, and heterogeneity of patient data, and thus we were not able to assess the impact of increase in time between care milestones on CI outcomes. Similarly, we were unable to assess the effect of virtual versus in person rehabilitation visits on speech and hearing outcomes. Despite these limitations, ample prior literature regarding the time‐sensitive nature of pediatric CI care provides evidence for the clinical significance of our findings.

## Conclusion

Overall, anticipatory measures that aimed to prevent delays in pediatric CI care during the COVID‐19 pandemic were successful in minimizing significant delays in care. Despite these efforts, however, patients with prelingual deafness experienced a significant delay between CI evaluation and surgery. Despite limited in‐person appointments, the total number of postoperative rehabilitation visits was not significantly impacted by COVID‐19, as many patients received rehabilitation virtually. Future studies may reveal whether speech and language outcomes have been impacted by delays in care and virtual rehabilitation among children implanted during the COVID‐19 pandemic.

## Author Contributions

**Kimberley S. Noij**, substantial contributions to conception and design, acquisition of data, and analysis and interpretation of data, drafting the article, final approval of the version to be published, and agreement to be accountable for all aspects of the work in ensuring that questions related to the accuracy or integrity of any part of the work are appropriately investigated and resolved; **Emily Y. Huang**, substantial contributions to acquisition of data, analysis and interpretation of data, revising the article critically for important intellectual content, final approval of the version to be published, and agreement to be accountable for all aspects of the work in ensuring that questions related to the accuracy or integrity of any part of the work are appropriately investigated and resolved. **Jonathan Walsh**, substantial contributions to analysis and interpretation of data, revising the article critically for important intellectual content, final approval of the version to be published, and agreement to be accountable for all aspects of the work in ensuring that questions related to the accuracy or integrity of any part of the work are appropriately investigated and resolved; **Francis X. Creighton**, substantial contributions to analysis and interpretation of data, revising the article critically for important intellectual content, final approval of the version to be published, and agreement to be accountable for all aspects of the work in ensuring that questions related to the accuracy or integrity of any part of the work are appropriately investigated and resolved. **Deepa Galaiya**, substantial contributions to analysis and interpretation of data, revising the article critically for important intellectual content, final approval of the version to be published, and agreement to be accountable for all aspects of the work in ensuring that questions related to the accuracy or integrity of any part of the work are appropriately investigated and resolved. **Stephen P. Bowditch**, substantial contributions to analysis and interpretation of data, revising the article critically for important intellectual content, final approval of the version to be published, and agreement to be accountable for all aspects of the work in ensuring that questions related to the accuracy or integrity of any part of the work are appropriately investigated and resolved. **C. Matthew Stewart**, substantial contributions to analysis and interpretation of data, revising the article critically for important intellectual content, final approval of the version to be published, and agreement to be accountable for all aspects of the work in ensuring that questions related to the accuracy or integrity of any part of the work are appropriately investigated and resolved. **Carolyn M. Jenks**, substantial contributions to conception and design, acquisition of data, analysis and interpretation of data, revising the article critically for important intellectual content, final approval of the version to be published, and agreement to be accountable for all aspects of the work in ensuring that questions related to the accuracy or integrity of any part of the work are appropriately investigated and resolved.

## Disclosures

### Competing interests

None.

### Funding source

None.
